# Cutaneous Reactions after COVID-19 Vaccines: Analysis of the Clinical and Histopathological Spectrum—Case Series and Review of the Literature

**DOI:** 10.3390/dermatopathology11010013

**Published:** 2024-03-14

**Authors:** Ursina Schmid, Jörg Galambos, Katrin Pfaltz, Ivan Hegyi, Salomé Courvoisier, Werner Kempf

**Affiliations:** 1 Kempf und Pfaltz Histologische Diagnostik, Affolternstrasse 56, CH-8050 Zurich, Switzerland; ursina.schmid2@uzh.ch (U.S.);; 2 Department of Dermatology, University Hospital Zurich, CH-8091 Zurich, Switzerland; 3 DermaPraxis, CH-8610 Uster, Switzerland

**Keywords:** cutaneous, adverse, COVID-19, vaccine, histology, clinicopathologic correlation

## Abstract

(1) Background: Various cutaneous adverse drug reactions (ADRs) are observed with the implementation of mRNA COVID-19 vaccines. To gain insight into the clinicopathologic features, we analyzed the correlation of histological and clinical data in 48 patients with these ADRs. (2) Methods: Single-center retrospective study in patients with ADRs after mRNA COVID-19 vaccination (mRNA-1273 and BNT162b2 vaccines). (3) Results: Distant generalized ADRs prevailed (91%), often appearing clinically as spongiotic dermatitis or maculopapular exanthema. Histopathological analysis revealed spongiotic changes (46%) and dermal superficial perivascular predominantly lymphocytic infiltrates (17%). Eosinophils were found in 66% of biopsies, neutrophils in 29%, and plasma cells only in 8% of biopsies. Most ADRs occurred after the second vaccine dose (44%). Histologically spongiotic changes were associated with clinical features of spongiotic dermatitis in only 50% of patients and maculopapular exanthema in the remaining patients. ADRs represented an aggravation of preexisting skin disease in 23% of patients. ADRs regressed within 28 days or less in 53% of patients and persisted beyond a month in the remaining patients. (4) Conclusions: Our study demonstrates a diverse spectrum of generalized ADRs, revealing correlations between histology and clinical features but also instances of divergence. Interestingly, in about half of our patients, ADRs were self-limited, whereas ADRs extended beyond a month in the other half.

## 1. Introduction

With the implementation of mRNA COVID-19 vaccines, various cutaneous adverse drug reactions (ADRs) are observed. As of September 2022, 67.7% of the world’s population had received at least one dose of an mRNA COVID-19 vaccine, with 12.58 billion doses administered globally [1]. The mRNA COVID-19 vaccines BNT162b2 (Comirnaty^®^; Pfizer–BioNTech) and mRNA-1273 (Spikevax^®^; Moderna) became available in December 2020 [2,3]. We performed our study in Switzerland, where 70% of the population had received at least one vaccination with either mRNA-1273 (43.9%), BNT162b2 (24.7%), or JNJ-78436735 (COVID-19 Vaccine Janssen; Johnson & Johnson) (0.7%) [4].

The application of mRNA COVID-19 vaccines can cause localized delayed-type hypersensitivity reactions (DTHRs) at the injection site (also referred to as “COVID arm”), as well as generalized rashes [5,6]. The most common ADRs reported after an mRNA COVID-19 vaccine are DTHRs at the site of injection as local reactions and disseminated reactions such as urticaria or morbilliform rashes, among several others [7,8,9,10,11,12,13,14,15,16].

As detailed data on the histopathological patterns of these cutaneous reactions are still sparse, the goal of this study was to characterize the histopathological aspects of the diverse ADRs after administration of the mRNA-1273 or BNT162b2 vaccines and to correlate the histopathological findings with clinical features. In comparison with previous studies, all cases could be analyzed for their histopathologic features in addition to clinical data. The knowledge of ADRs after mRNA COVID-19 vaccines will be of benefit in the near future when the use of mRNA vaccines is expanded for other indications, such as the treatment of malignancies or prevention of other infectious diseases.

## 2. Materials and Methods

We conducted a retrospective study on ADRs in patients who were vaccinated with either the BNT162b2 or mRNA-1273 vaccine. We included only cases in which skin biopsies of ADRs had been taken and in which clinical and histopathological findings could be analyzed. Tissue sections of 3–4 micrometer thickness—fixed in formalin (4%) and embedded in paraffin—were stained with hematoxylin and eosin (H&E), periodic-acid-Schiff (PAS) stain, and Giemsa stain. All biopsies were reevaluated by one of the authors (WK) for the presence of epidermal changes (such as acanthosis, spongiosis, hyper- or parakeratosis with or without inclusions of neutrophils, and acantholysis), interface changes (including vacuolization in the junctional zone, apoptotic keratinocytes, exocytosis of lymphocytes), pattern and composition of the dermal infiltrate (superficial and/or deep perivascular predominantly lymphocytic infiltrates), and subcutaneous infiltrates (septal vs. lobular, with or without vasculitis). In addition, all biopsies were evaluated for the presence of eosinophils, neutrophils, and plasma cells. Mast cells were assessed by evaluating Giemsa stains. The histopathological findings were assigned to one of the following histopathological reaction patterns: spongiotic, superficial perivascular predominantly lymphocytic without epidermal changes, psoriasiform, lichenoid, non-lichenoid interface changes, urticarial, vasculitis, panniculitis, bullous, acantholytic and dyskeratotic, pustular, folliculitis, and pseudolymphomatous. To assess the clinical data, a questionnaire was sent to the treating physicians asking for the clinical morphology of the cutaneous reactions, clinical images, as well as the type of mRNA COVID-19 vaccine applied, and—if available—the latency between administration of the vaccine and onset of ADRs. We compared the clinical patterns with the histopathological patterns and categorized the reactions based on clinicopathological correlation. Furthermore, we assessed the clinical and histopathological features to see if they fit the criteria for V-REPP and, if so, under which category, as defined by McMahon et al. [16] (Table 1). The term “V-REPP” was introduced by McMahon et al. as a vaccine-related eruption of papules and plaques and defined as “*discrete edematous papules, some with central vesiculation and crusting and its histopathology including spongiotic dermatitis as robust intercellular edema with intraepidermal vesicles, papillary dermal edema, and dermal eosinophils. Interface changes may or may not be present*.” [16].

## 3. Results

### 3.1. Patients

Out of the 111 patients contacted for their written informed consent, 48 agreed to participate in our study. This group included a total of 24 men and 24 women. The median age was 66.3 years (ranging from 24 to 90 years). Out of 48 patients, only 2 (4%) stated they had experienced a SARS-CoV-2-infection months before receiving the vaccine

### 3.2. Vaccine Types and Latency

A total of 35 patients (73%) received the mRNA-1273 vaccine and 12 (25%) received the BNT162b2 vaccine, while only 1 person (2%) received both vaccines. Fourteen (29%) biopsies were taken from lesional skin occurring after the first vaccine, 21 (44%) from lesional skin occurring after the second vaccine, and 5 (10%) from lesional skin occurring after the third or fourth vaccinations. In 14 patients (29%), the reaction appeared after the first vaccination only. ADRs after the first dose took 10 days to develop on average, ranging from 1 day up to 28 days. In 21 patients (44%), the reaction occurred only after the second vaccine and manifested after 8.7 days on average (range: 1–21 days). Five patients (10%) developed a cutaneous reaction after the first and second doses.

### 3.3. Distribution of Cutaneous Reactions

Out of 48 patients (85%), 41 developed distant reactions on two or more body areas (distant disseminated). Three patients (6%) exhibited localized reactions that were located on body areas other than the vaccinated arm (distant localized). The remaining four patients (9%) showed localized ADRs at the vaccination site.

### 3.4. Cutaneous Reactions—Spectrum of Clinical Manifestations

**Figure 1:**
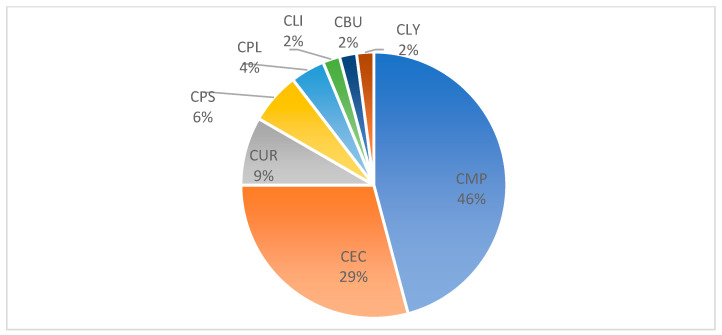
Distribution (%) of clinical patterns after vaccination with either mRNA-1273 or BNT162b2. Abbreviations: maculopapular exanthema (CMP), spongiotic dermatitis (syn. eczema) (CEC), urticarial (CUR), psoriasis-like (CPS), plaque-like (CPL), lichenoid (CLI), bullous (CBU), and pseudolymphomatous (CLY).

**Figure 2:**
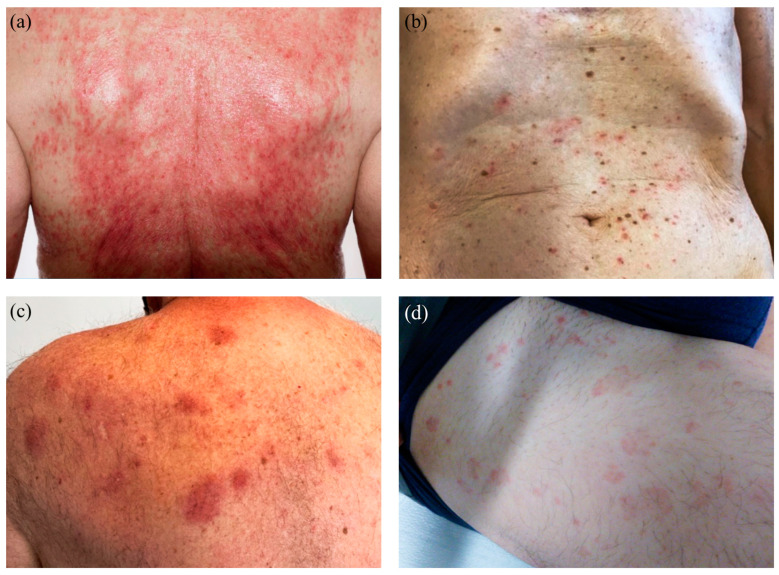
Clinical manifestations of COVID-vaccine-associated adverse reactions (ADRs). (**a**) Maculopapulous confluent on the back. (**b**) Maculopapulous exanthema located on the frontal torso. (**c**) Erythematous plaques on the left upper back. (**d**) Wheals located on the thigh, frontal and lateral.

Clinically, skin lesions were reported as maculopapular exanthema (*n* = 22, 46%), spongiotic dermatitis (*n* = 14, 29%), urticarial (*n* = 4, 9%), psoriasis-like (*n* = 3, 6%), and plaque-like (*n* = 2, 4%) reactions as well as lichenoid (2%), bullous (2%), and pseudolymphomatous (2%) patterns in one patient each (Figure 1 and Figure 2). Out of 48 patients, 14 (29%) clinically showed spongiotic dermatitis after mRNA-1273 (*n* = 9) and BNT162b2 (*n* = 4). Three patients experienced a reaction after the first vaccine only, five after the second only, two after the first and second doses, two after the third vaccine only, and two after all three doses. One patient received mRNA-1273 for their first vaccine dose and then switched to BNT162b2 for the second and third doses but experienced a clinically spongiotic reaction after all three doses. A total of 8 patients clinically presenting with maculopapular exanthema developed the reaction after the first vaccine only, 11 after the second vaccine only, 2 after the third vaccination only, and 1 patient after both the first and second vaccinations. All these ADRs were skin diseases of new onset. For this study, we use the term “spongiotic dermatitis” synonymously with the term “eczema”.

### 3.5. Cutaneous Reactions—Spectrum of Histopathological Features

**Figure 3:**
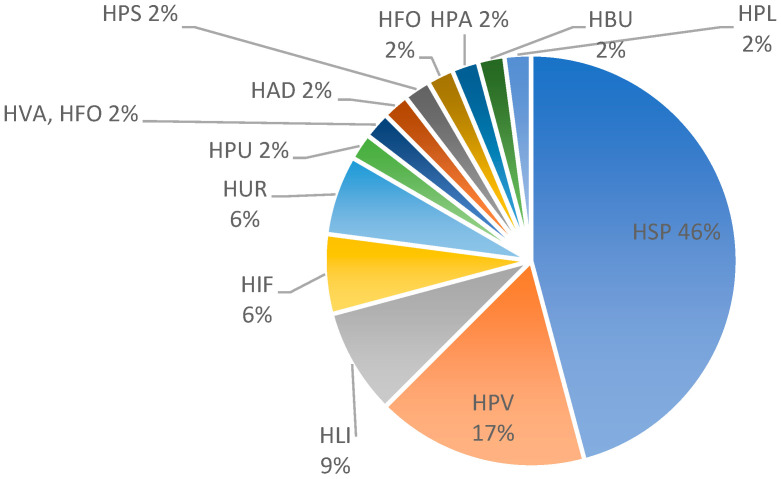
Distribution (%) of histological patterns after vaccination with either mRNA-1273 or BNT162b2. Abbreviations: spongiotic (HSP), superficial perivascular predominantly lymphocytic without epidermal changes (HPV), psoriasiform (HPS), lichenoid (HLI), non-lichenoid interface changes (HIF), urticarial (HUR), vasculitis (HVA), panniculitis (HPA), bullous (HBU), acantholytic and dyskeratotic (HAD), pustular (HPU), folliculitis (HFO), and pseudolymphomatous (HPL).

**Figure 4:**
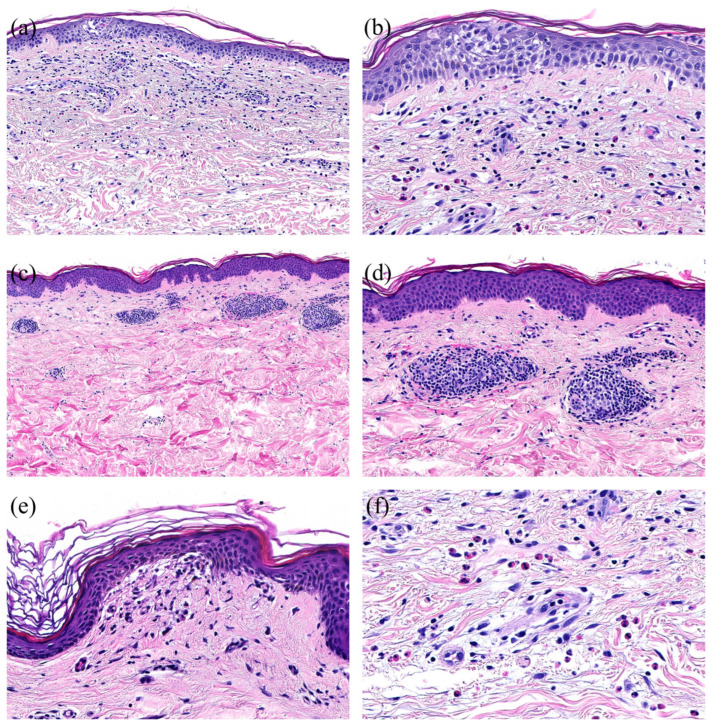
Histopathological patterns of COVID-vaccine-associated cutaneous adverse reactions (ADRs). (**a**) Spongiotic pattern with focal spongiosis of the epidermis and perivascular predominantly lymphocytic infiltrate (H&E, magnification ×20). (**b**) Spongiotic pattern (detail) with focal spongiosis of the epidermis and exocytosis of lymphocytes in the spongiotic areas (H&E, magnification ×200). (**c**) Perivascular pattern with perivascular predominantly lymphocytic infiltrate in the upper dermis in the absence of epidermal changes (H&E, magnification ×20). (**d**) Perivascular pattern (detail) with sleeve-like perivascular predominantly lymphocytic infiltrate in the upper dermis (H&E, magnification ×200). (**e**) Interface pattern with vacuolization in the junctional zone (H&E, magnification ×200). (**f**) High number of eosinophils in the infiltrate (H&E, magnification ×200).

The most common histopathological patterns were spongiotic (*n* = 22; 46%), followed by superficial perivascular predominantly lymphocytic infiltrate (in the absence of epidermal changes) (*n* = 8; 17%), and lichenoid pattern (*n* = 4; 9%). Less common histological patterns comprised an urticarial pattern (*n* = 3; 6%), interface changes (*n* = 3; 6%), and additional patterns, each represented by one biopsy (*n* = 1, 2%): psoriasiform, pustular, bullous, vasculitis, panniculitis, pseudolymphomatous, folliculitis, and acantholytic and dyskeratotic changes (Figure 3 and Figure 4). In most cases, the perivascular infiltrates were cuffed and confined to the superficial vascular plexus as representatively depicted in Figure 4. The pseudolymphomatous infiltrates simulated nodular T- or B-cell lymphoma and did not resemble mycosis fungoides or lymphomatoid papulosis. The bullous reaction resembles bullous pemphigoid. Spongiosis was found in some of the spongiotic dermatitis cases but was usually only focal. Neutrophils were present in 14 biopsies (29%), and plasma cells in 4 biopsies (8%). Mast cells assessed in Giemsa stains were identified in the HSP and HPV patterns, but we did not see any increase in the number of mast cells. Eosinophils were found in a total of 32 biopsies (66%), including spongiotic pattern (16 of 20 patients; 80%), superficial perivascular predominantly lymphocytic infiltrate (5 of 8 patients; 63%), urticarial pattern (3 of 3 patients; 100%), lichenoid pattern (2 of 4 patients; 50%), and interface changes (2 of 3 patients; 66%) and in one patient each with folliculitis, bullous, and acantholytic and dyskeratotic changes and a combination of vasculitis and folliculitis. Based on the number of eosinophils found in one high power field (HPF), we divided the biopsies with eosinophils into three groups (E1-E3): E1, in which one eosinophil per HPF was detected, was the biggest group with 14 biopsies (44%); E2 (*n* = 12; 37%) when 2–5 eosinophils per HPF were detected; and E3 (*n* = 6; 19%) for >5 eosinophils per HPF.

### 3.6. Correlation of Histological Patterns with Clinical Presentations

The correlation of histological spongiotic changes with the clinical features revealed that only 11 of 22 (50%) patients also clinically showed spongiotic dermatitis. Remarkably, 10 of the other patients (45%) clinically exhibited maculopapular exanthema, and 1 patient clinically showed plaques (5%). The patients with clinically spongiotic dermatitis (*n* = 14) displayed spongiotic changes (*n* = 11; 79%), dermal superficial perivascular predominantly lymphocytic infiltrates (*n* = 1; 7%), psoriasiform (*n* = 1; 7%), and vacuolar pattern of interface changes (*n* = 1; 7%) in their biopsies. The second most common histological group was characterized by superficial perivascular predominantly lymphocytic without epidermal changes (*n* = 8; 17% of all patients). Seven of these eight patients (87%) clinically manifested with maculopapular exanthema and one patient (13%) with clinically spongiotic dermatitis. The 22 patients (46% of all patients) with clinical presentation of maculopapular exanthema showed histologically spongiotic changes (*n* = 10; 45.5%), while a superficial perivascular predominantly lymphocytic infiltrate was found in 7 patients (32%). Other histological patterns were interface changes (4.5%), pustular (4.5%), lichenoid (4.5%), acantholytic and dyskeratotic (4.5%), and folliculitis (4.5%) in one patient each. Of the patients, 19 (86%) received mRNA-1273, and 3 (14%) were vaccinated with BNT162b2. Applying the criteria for *V-REPP* of McMahon et al., V-REPP was found in 23 patients (48%). The robust form accounted for 20/23 biopsies (87%), whereas the moderate (*n* = 0) and mild (*n* = 3/23; 13%) forms of V-REPP were rarely observed. Of these patients with V-REPP, 20 (87%) were vaccinated with mRNA-1273, whereas the remaining 3 patients (13%) were vaccinated with BNT162b2. Eight patients (35%) experienced this reaction after the first vaccine only, eight (35%) after the second vaccine only, and four (17%) after the third vaccine only. The cutaneous reaction appeared between 1 and 9 days in 12 patients (52%), between 10 and 14 days in 5 patients (22%), and between 21 and 28 days in 6 patients (26%).

### 3.7. New-Onset Cutaneous Reaction vs. Aggravation of Preexisting Skin Disease

Eleven patients (23%) experienced an aggravation of preexisting skin disease, including four of the patients with clinical spongiotic dermatitis, one of the V-REPP patients, one patient with a psoriasiform reaction, one with a lichenoid pattern, all three of the patients with urticaria, and one patient with bullous pemphigoid. Thirty-seven patients (77%) experienced a new-onset skin reaction after an mRNA COVID-19 vaccine.

### 3.8. Duration of Cutaneous Reactions

Information about the duration of the ADRs was obtained from 45 patients (94%). In most patients (24/45; 53%), ADRs regressed within 28 days or less (range 1–28 days), whereas ADRs lasted more than 28 days in 21 patients (21/45; 47%).

## 4. Discussion

The results of our study highlight the broad spectrum of clinical and histopathological presentations of ADRs in patients receiving mRNA COVID-19 vaccines. In the generalized form of ADRs, maculopapular exanthema and spongiotic dermatitis were the most common clinical manifestations accounting for 75% of all generalized cutaneous reactions. The incidence of urticarial reaction in our study seems to be slightly lower than in other studies [6,11,17]. Most patients (three out of four; 75%) with a clinically urticarial rash had preexisting urticaria triggered by the vaccination.

Histologically, the spongiotic pattern and the superficial perivascular predominantly lymphocytic pattern were the most common, accounting for more than half of the biopsies. In contrast, interface changes with a vacuolar or lichenoid pattern were less common.

Interestingly, the spongiotic pattern was the histological correlate of most cases presenting clinically with maculopapular exanthema, and fewer showed dermal superficial perivascular predominantly lymphocytic changes as the major histological finding.

In the study by Magro et al., as well as in our study, only half of the cases with histologically spongiotic features were also clinically described as a spongiotic reaction [10,17,18,19,20,21,22,23,24]. Conversely, not all cases presenting clinically with spongiotic dermatitis also showed histologically spongiotic changes. These results demonstrate that clinical presentation and histologic features may diverge in individual cases. Dermatologists and dermatopathologists should be aware of this potential discrepancy.

A large series with 58 biopsies of ADRs to mRNA COVID-19 vaccines was analyzed by McMahon et al. [16]. These biopsies, however, represented only 7% of all ADRs recorded in their registry. Spongiotic changes were the most common histopathological reaction and clinically correlated with papules, plaques, or pityriasis rosea-like eruptions. For this constellation, the acronym “V-REPP” was proposed by McMahon et al. with three different grades (robust, moderate, and mild). Almost half of our patients showed clinical features compatible with V-REPP, which thereby represented the most common ADR in our series when applying the criteria of McMahon et al. Within this group, the robust form accounted for almost 90% of the biopsies, whereas the moderate and mild forms were rarely observed. V-REPP, however, is clinically and histologically heterogeneous. The histological hallmark of V-REPP is spongiosis, associated with interface changes of various degrees, ranging from significant spongiosis with intraepidermal vesicle formation and minimal or no interface changes (robust V-REPP) to pityriasiform spongiosis (moderate V-REPP) or minimal spongiosis with more prominent interface changes (mild V-REPP). Interestingly, the spongiotic pattern was not associated with interface changes in our cohort. Most patients in our cohort with histologically spongiotic changes might be classified as V-REPP when applying the criteria of McMahon et al. However, we classified patients with clinical features of spongiotic dermatitis and histological spongiotic changes synoptically as spongiotic dermatitis, and not as a robust form of V-REPP. This difference may explain the higher rate of spongiotic dermatitis in our cohort. A similarly high rate of clinical spongiotic reaction was also observed by Kroumpouzos et al. [17]. Since the vast majority of our patients with clinically and/or histologically spongiotic changes had received the mRNA-1273 vaccine, these findings may indicate that mRNA-1273 more commonly induces spongiotic dermatitis than other vaccines.

Histological analysis demonstrated that eosinophils were a consistent feature of all reaction patterns and were found in two-thirds of the biopsies. Eosinophils were present in the majority of the HSP reaction patterns and in over half of the HPV reaction patterns, similar to other studies [10,18]. There was an admixture of mast cells in all reactions, but their number did not increase. However, it cannot be excluded that the mast cells involved in vaccine-related reactions require different staining techniques to be highlighted [19]. Plasma cells were only found in a small subset of biopsies.

The localized form of ADRs is characterized by erythema and swelling at the injection site (“COVID arm”). This delayed-type hypersensitivity reaction (DHSR) is histologically characterized by subtle and only very focal epidermal changes with spongiosis and exocytosis of a few lymphocytes and sleeve-like perivascular infiltrates with features of erythema annulare centrifugum [5,7,20]. In our four patients with COVID arm, we noticed similar findings.

In more than 70% of the patients in our cohort, the ADRs were a new-onset skin disease and showed a self-limiting course. A subset of ADRs after mRNA COVID-19 vaccination, however, represented an aggravation of a previously existing inflammatory skin disorder such as atopic dermatitis. This reflects the result of immune activation, which is not specific to mRNA COVID-19 vaccines, as aggravation or relapse of preexisting skin diseases after vaccination, in general, was observed [17,21,22,23,24,25]. An interesting question to study would be whether the aggravation of preexisting skin diseases after vaccination would differ if patients were under active treatment for their underlying disease at the time of vaccination. Furthermore, it would be of interest to clarify whether patients with a preexisting skin disease had an active underlying skin disease that worsened after vaccination, or if some patients with inactive skin disease showed a recurrence of the skin disease after vaccination. Further studies need to be conducted to determine if patients with preexisting skin diseases or atopy do have a higher probability of developing ADRs after mRNA vaccines [17,24].

In the literature, heterogenous data on the occurrence of ADRs after the first or subsequent doses was reported [6,16,17,26,27]. The ADRs in our study most frequently occurred after the second dose, which contrasts with the 2.3% and 26.9% of ADRs after the second dose in previous studies [11,17]. The differences may be related to the predominance of the mRNA-1273 vaccine given to the patients in our series. In our study, one-third of our patients who developed an ADR after the first dose experienced an ADR after the second dose as well. However, it is unclear if, in the remaining two-thirds of patients with an ADR after the first dose, the patients refused to have the second dose or if they simply did not have an ADR develop again after the second dose. Whereas “COVID arm” usually resolves within 1 or 2 weeks, generalized ADRs lasted in many patients in our cohort for more than 28 days and in some patients even longer than 6 months. This duration is longer than the data reported by other groups, which observed regression of ADRs in most patients within 14 days [5,6,28,29]. In V-REPP, however, longer duration times of up to 49 days and rarely up to 90 days were reported.

Our cohort differs in two epidemiologic aspects from other studies. In our cohort, the gender distribution is equal, whereas other studies reported a higher number of ADRs in women [6,9,17,23,26,28]. Second, the vast majority of our patients (75%) had received the mRNA-1273 vaccine, as in Switzerland far more people in general were vaccinated with mRNA-1273 [4]. McMahon et al. also stated that they received more reports on cutaneous reactions after the mRNA-1273 vaccine than after the BNT162b2 vaccine [6].

Pathogenetically, V-REPP seems to be initiated by Th1-cells, whereas Th2-cells trigger the development of spongiotic dermatitis [10]. The mode of action may differ between various vaccines, but increased interferon-gamma produced by vaccine-induced receptor binding domain-specific CD8^+^ and CD4^+^ T cells seems to play a crucial role in the mediation of vaccine-related ADRs [10,30].

In conclusion, we found a wide spectrum of cutaneous ADRs in our patients. Although the histological pattern correlated in many patients with the clinical manifestation, discrepant findings could be observed, especially regarding spongiotic changes. The majority of ADRs were self-limited; however, a few ADRs lasted for more than one month. The vast majority of ADRs were new-onset reactions, while the remaining ADRs represented aggravation of a preexisting skin disease. This knowledge of the spectrum of cutaneous ADRs after vaccination with mRNA vaccines will be helpful for dermatologists and other specialties in counseling patients regarding these vaccinations.

## Figures and Tables

**Table 1:** Overview of patient data including age, gender, vaccine type, exacerbation/new onset, and latency, as well as clinical and histological patterns.

Study Number	Gender	Age	Exacerbation/New Onset	Vaccine	L (Local)	D (Distant)	Eosinophil	Neutrophil	Plasma Cells	1. Dose, Latency	2. Dose, Latency	3. Dose, Latency	4. Dose, Latency	Clinical Pattern	Histological Pattern	Comparison with McMahon et al.
1	m	59	new-onset	mRNA-1273		DD	E0	N1	P0		1 d			CEC	HSP	Robust V-REPP
2	m	37	exacerbation	mRNA-1273		DD	E1	N0	P0	2 d	1.5 d			CEC	HSP	Robust V-REPP
3	w	67	exacerbation	mRNA-1273		DD	E2	N0	P0		1 d			CEC	HSP	Robust V-REPP
4	w	63	new-onset	mRNA-1273		DD	E2	N0	P0			14 d		CEC	HSP	Robust V-REPP
5	w	56	new-onset	BNT162b2		DD	E3	N0	P0	1 d				CEC	HSP	Robust V-REPP
6	w	54	new-onset	mRNA-1273		DD	E0	N0	P0	1 d	1 d	1 d		CEC	HSP	Robust V-REPP
7	w	59	new-onset	mRNA-1273 /BNT162b2		DD	E0	N2	P0	1 d (mRNA-1273)	1.5 d (BNT162b2)	1 d (BNT162b2)		CEC	HSP	Robust V-REPP
8	m	72	new-onset	mRNA-1273		DD	E1	N1	P0	9 d				CEC	HSP	Robust V-REPP
9	w	79	exacerbation	mRNA-1273		DD	E3	N0	P0		7 d			CEC	HSP	Robust V-REPP
10	w	32	exacerbation	mRNA-1273		DD	E1	N0	P0	14 d				CEC	HSP	Robust V-REPP
11	w	85	exacerbation	BNT162b2		DD	E0	N2	P0	2 d	2 d			CEC	HPS	other
12	m	58	exacerbation	mRNA-1273		DD	E2	N0	P0			10 d		CEC	HIF	mild V-REPP
13	m	62	new-onset	BNT162b2		DD	E0	N0	P0		14 d			CEC	HPV	dermal hypersensitivity
14	m	81	new-onset	BNT162b2		DD	E1	N0	P1			28 d		CMP	HSP	Robust V-REPP
15	w	82	new-onset	mRNA-1273		DD	E2	N2	P1		5.5 d			CMP	HSP	Robust V-REPP
16	m	38	new-onset	mRNA-1273		DD	E1	N0	P0		21 d			CMP	HSP	Robust V-REPP
17	m	52	new-onset	mRNA-1273		DD	E1	N1	P0	21 d				CMP	HSP	Robust V-REPP
18	m	70	new-onset	mRNA-1273		DD	E2	N0	P0	7 d				CMP	HSP	Robust V-REPP
19	w	70	new-onset	BNT162b2		DD	E0	N0	P0	28 d				CMP	HSP	Robust V-REPP
20	w	80	new-onset	mRNA-1273		DD	E2	N2	P0		5 d			CMP	HSP	Robust V-REPP
21	w	70	new-onset	mRNA-1273		DD	E1	N0	P0		21 d			CMP	HSP	Robust V-REPP
22	m	78	new-onset	mRNA-1273		DD	E1	N2	P1	3 d				CMP	HPV	dermal hypersensitivity
23	w	70	new-onset	BNT162b2		DD	E2	N0	P0		2 d			CMP	HPV	dermal hypersensitivity
24	w	74	new-onset	mRNA-1273		DD	E1	N0	P0		7 d			CMP	HPV	dermal hypersensitivity
25	m	74	new-onset	mRNA-1273		DD	E3	N0	P0		4 d			CMP	HPV	dermal hypersensitivity
26	m	44	new-onset	mRNA-1273		DD	E0	N0	P0		14 d			CMP	HPV	dermal hypersensitivity
27	m	82	new-onset	mRNA-1273		DD	E0	N1	P0	6 d				CMP	HPU	other
28	m	75	new-onset	mRNA-1273		DD	E0	N0	P0	21 d				CMP	HIF	mild V-REPP
29	m	50	new-onset	mRNA-1273		DL	E1	N0	P0		17 d			CMP	HFO	other
30	w	81	new-onset	mRNA-1273		DL	E0	N0	P0	10 d				CMP	HLI	LP-like
31	w	81	new-onset	mRNA-1273		DD	E3	N2	P0			14 d		CMP	HSP	Robust V-REPP
32	m	41	new-onset	mRNA-1273		DD	E0	N2	P0		7 d			CMP	HSP	robust V-REPP
33	m	33	new-onset	mRNA-1273		DD	E1	N0	P0	10 d				CUR	HIF	mild V-REPP
34	w	81	exacerbation	mRNA-1273		DD	E0	N0	P0	5.5 d	6.5 d			CLI	HLI	LP-like
35	w	73	exacerbation	BNT162b2		DD	E2	N0	P0		5 d			CUR	HUR	urticaria
36	m	39	exacerbation	mRNA-1273		DD	E2	N0	P0					CUR	HUR	urticaria
37	w	24	exacerbation	mRNA-1273		DD	E2	N2	P0	3 d				CUR	HUR	urticaria
38	w	87	new-onset	mRNA-1273	L		E3	N0	P0	6 d				CPL	HSP	dermal hypersensitivity
39	m	77	new-onset	BNT162b2	L		E0	N0	P0		15 d			CEC	HSP	dermal hypersensitivity
40	w	81	new-onset	mRNA-1273	L		E2	N0	P0	7 d				CMP	HPV	COVID-Arm
41	w	67	new-onset	mRNA-1273	L		E0	N0	P1	5.5 d	8.5 d			CMP	HPV	dermal hypersensitivity
42	m	81	new-onset	mRNA-1273		DD	E3	N0	P0		1 d			CMP	HAD	M. Grover
43	m	71	new-onset	BNT162b2		DL	E1	N0	P0				6 d	CPL	HVA, HFO	vaskulitis
44	m	85	exacerbation	mRNA-1273		DD	E2	N3	P0	14 d	2 d			CBU	HBU	Bullous pemphigoid-like
45	w	90	new-onset	mRNA-1273		DD	E1	N0	P0		3.5 d			CPS	HLI	LP-like
46	m	70	new-onset	BNT162b2		DD	E1	N0	P0		7 d			CPS	HLI	LP-like
47	w	77	new-onset	BNT162b2		DD	E0	N2	P0		weeks			CPS	HPA	other
48	m	69	new-onset	BNT162b2		DD	E0	N0	P0		16 d			CLY	HPL	other

Abbreviations: maculopapular exanthema (CMP), spongiotic dermatitis (syn. eczema) (CEC), urticarial (CUR), psoriasis-like (CPS), plaque-like (CPL), lichenoid (CLI), bullous (CBU), and pseudolymphomatous (CLY). Spongiotic (HSP), superficial perivascular predominantly lymphocytic without epidermal changes (HPV), psoriasiform (HPS), lichenoid (HLI), non-lichenoid interface changes (HIF), urticarial (HUR), vasculitis (HVA), panniculitis (HPA), bullous (HBU), acantholytic and dyskeratotic (HAD), pustular (HPU), folliculitis (HFO), and pseudolymphomatous (HPL).

## Data Availability

The data presented in this study are available on request from the corresponding author. The data are not publicly available due to patient confidentiality.
